# Signal Detection of Adverse Drug Reactions of Cephalosporins Using Data from a National Pharmacovigilance Database

**DOI:** 10.3390/ph14050425

**Published:** 2021-05-02

**Authors:** Jung-Yoon Choi, Jae-Hee Choi, Myeong-Gyu Kim, Sandy-Jeong Rhie

**Affiliations:** 1 Graduate School of Converging Clinical & Public Health, Ewha Womans University, Seoul 03760, Korea; cjyjy0512@gmail.com; 2 Division of Life and Pharmaceutical Sciences, Ewha Womans University, Seoul 03760, Korea; pddari@naver.com; 3 Department of Pharmacy, Konkuk University Hospital, Seoul 05030, Korea; 4 College of Pharmacy and Graduate School of Pharamceutical Sciences, Ewha Womans University, Seoul 03760, Korea

**Keywords:** adverse drug reaction, cephalosporin, KIDS KAERS database (KIDS-KD), pharmacovigilance, signal

## Abstract

This case-non-case study aims to detect signals not currently listed on cephalosporin drug labels. From 2009 to 2018, adverse event (AE) reports concerning antibacterial drugs (anatomical therapeutic chemical (ATC) code J01) in the Korea Adverse Events Reporting System (KAERS) database were examined. For signal detection, three indices of disproportionality, proportional reporting ratio (PRR), reporting odds ratio (ROR), and information component (IC), were calculated. The list of signals was compared with ADRs on the drug labels from the United States, United Kingdom, Japan, and South Korea. A total of 163,800 cephalosporin–AE combinations and 72,265 all other J01–AE combinations were analyzed. This study detected 472 signals and 114 new signals that are not included on the drug labels. Cefatrizine–corneal edema (PRR, 440.64; ROR, 481.67; IC, 3.84) and cefatrizine–corneal ulceration (PRR, 346.22; ROR, 399.70; IC, 4.40) had the highest PRR, ROR, and IC among all signals. Additionally, six serious AEs that were not listed on drug labels such as cefaclor-induced stupor (ten cases) and cefaclor-induced respiratory depression (four cases) were found. Detecting signals using a national pharmacovigilance database is useful for identifying unknown ADRs. This study identified signals of cephalosporins that warrant further investigation.

## 1. Introduction

Cephalosporins are one of the most commonly used antibiotics in clinical practice [1]. Cephalosporins are generally considered safe; but allergic reactions, renal dysfunction, hepatic dysfunction, and seizures have occurred as cephalosporin-class adverse reactions and are listed on the drug labels. These adverse drug reactions (ADRs) are responsible for morbidity and mortality and represent a significant burden to both the affected patient and to the health care system [2,3].

ADRs are usually detected during clinical trials. However, short testing periods and small numbers of subjects in clinical trials hinder identification of all ADRs before marketing [4]. For this reason, investigation of post-marketing adverse events (AEs) is necessary to ensure the safety of the drug. In particular, rare (1 in 1000) or very rare (1 in 10,000) serious ADRs can only be observed after a large number of patients have been administered the drug [5]. AE reporting systems are widely used and cost-effective methods for collecting post-marketing AEs [5]. Spontaneous AE reports from pharmaceutical companies, national and international pharmacovigilance centers, or regulatory authorities are collected in national pharmacovigilance databases [5]. The Korea Institute of Drug Safety & Risk Management (KIDS) developed the Korea Adverse Events Reporting System (KAERS) database (KIDS-KD) to collect and manage AE reports [6]. Appropriate statistical analysis of AE reports that were collected in the database enables detection of signals of unknown ADRs [7]. A signal is defined as the information implying a known or unknown association among drugs and AEs that warrants further investigation [8].

Large-scale studies have investigated AEs of cephalosporins from hospital data or AE reporting systems [2,3,9,10]. However, these studies only descriptively analyzed the frequency of AEs and did not detect signals using statistical analysis. Some studies analyzed the cephalosporin-class adverse reactions (anaphylaxis, renal dysfunction, hepatic dysfunction, and Clostridium difficile infection) that have already been well-documented [11,12,13,14,15].

The aim of this study was to detect signals that are not currently listed on drug labels of cephalosporins through analyzing AE reports in the KIDS-KD.

## 2. Results

### 2.1. Characteristics of AE Reports

There were 163,800 cephalosporin–AE combinations in 110,965 reports and 72,265 all other J01–AE combinations in 45,780 reports. Characteristics of AE reports concerning cephalosporins and all other J01 are presented in Table 1. The AEs of cephalosporins were frequently reported in women (55.74%) and patients aged 51–60 years (18.50%). Serious AEs of cephalosporins accounted for 6.76%, most of which caused hospitalization or prolonged hospitalization (48.71%).

### 2.2. Cephalosporin-Induced AEs

The 25 most commonly reported AEs of cephalosporins are presented in Table 2. Cephalosporin-induced AEs were mainly skin and appendage disorders and gastrointestinal system disorders. The most frequently reported AEs were nausea (12.44%), followed by rash (11.99%), pruritus (9.25%), urticaria (7.76%), and vomiting (6.13%).

### 2.3. Signal Detection

A total of 472 cephalosporin-AE combinations satisfied all three criteria of the signal. Among them, 114 new signals that are not included on the drug labels were found. The cephalosporin–AE combinations and values of proportional reporting ratio (PRR), reporting odds ratio (ROR), and Bayesian confidence propagation neural networks of information components (IC) are presented in Table S1. Cefatrizine-corneal edema (PRR, 440.64; ROR, 481.67; IC, 3.84) and cefatrizine-corneal ulceration (PRR, 346.22; ROR, 399.70; IC, 4.40) had the highest PRR, ROR, and IC among all signals.

A total of 78 serious AEs were found among 472 signals. Among these 78, six serious AEs were not included on the drug labels (Table 3). In particular, there were cefaclor-induced stupor (ten cases) and cefaclor-induced respiratory depression (four cases).

First- and second-generation cephalosporins had significantly higher ROR in four system organ classes (SOCs) compared to all other J01 (Table 4). In particular, the ROR values for respiratory system disorders were high, 4.26 (95% confidence interval (CI), 2.64–6.90) and 5.46 (95% CI, 3.66–8.13), for the first- and second-generation cephalosporins, respectively. The third-generation cephalosporin had a significantly higher ROR only for respiratory system disorders (ROR, 1.68; 95% CI, 1.05–2.69). However, the fourth-generation cephalosporin had lower ROR values for serious AEs compared to other systemic antibiotics. For the general disorders, the ROR value was significantly lower (ROR, 0.29; 95% CI, 0.15–0.56) for the fourth-generation cephalosporin. However, the fourth-generation cephalosporin had significantly higher ROR values for central and peripheral nervous system disorders (ROR, 4.43; 95% CI, 2.34–8.40).

## 3. Discussion

In this study, AE reports concerning cephalosporins were investigated and signals were detected through disproportionality analysis. As a result, 114 signals and six serious ADRs that were not included on the drug labels were found. To our knowledge, this is the first study that detected new signals through disproportionality analysis of a national pharmacovigilance database.

Allergic reactions were previously the most commonly reported cephalosporin-related AE with a frequency of approximately 1 to 5% [16,17]. These reactions are mainly manifested as skin symptoms such as rash, pruritus, urticaria, and angioedema [16,17,18,19]. In addition, severe cutaneous adverse reactions (SCARs), such as acute generalized exanthematous pustulosis (AGEP), Stevens-Johnson syndrome (SJS)/toxic epidermal necrolysis (TEN), and drug rash with eosinophilia and systemic symptoms (DRESS) can also be caused by cephalosporin [20,21,22]. Skin symptoms accounted for approximately one-third of the cephalosporin-induced AEs in the present study. The results that the organs most commonly affected were the skin and appendages were consistent with those of other studies [3,9,23].

A national pharmacovigilance database enables detection of unknown ADRs in addition to known ADRs. The KIDS-KD has been successfully used to detect signals of several drugs including imipenem and quinolone antibiotics [24,25]. In this study, we were able to detect signals of cephalosporins. For example, cefatrizine, a first-generation cephalosporin used in East and Southeast Asia, was found to be associated with corneal ADRs. Cefatrizine-induced corneal ADRs have never been reported in the literature. This confirms the usefulness of a national pharmacovigilance database for detecting unidentified ADRs.

Most ADRs of cephalosporins are mild, self-limiting, and resolve with discontinuation of the drug [16]. However, serious ADRs can affect morbidity and mortality. Thus, this study focused on serious AEs. Some of the reported serious AEs such as anaphylactic shock are well-documented ADRs of cephalosporins. The present study identified six serious AEs that were not listed on the drug labels. Among the AEs, cefaclor-induced stupor is the most commonly reported. Among cephalosporins, stupor is listed only on the drug label of cefepime. Cefepime, a fourth-generation cephalosporin, can access the cerebral spinal fluid [26]. Cefepime has been associated with the induction of seizures and encephalopathy. The mechanism proposed for this induction is the competitive inhibition of γ-aminobutyric acid (GABA) A receptors [27]. Other cephalosporin antibiotics can also cause serious central nervous system toxicities [10]. The risk is particularly high if the drug accumulates in the body due to renal failure [10,28]. Although stupor is not listed as an ADR of cefaclor, caution is needed.

This study has some limitations similar to those of other studies using a national pharmacovigilance database. First, since not all AEs are reported, the occurrence of AEs may be underestimated due to underreporting. Second, unlike information-rich hospital data, data from the KIDS-KD omitted information about the clinical status of patients, for example kidney and liver function. This limits the adjustment to the risk factors of ADRs. Third, some information about concomitant drugs that can affect specific AEs was also missing. The possibility of under-reporting of concomitant drugs is inevitable in studies with KIDS-KD. Fourth, the preferred term (PT) code may not be stated explicitly on the drug label. The difference in ADR terms was a barrier in evaluating whether a signal is recorded on the drug label. Moreover, an unlisted signal can be a symptom of a listed ADR. Last, some signals such as bronchitis and pharyngitis may be incorrectly detected. These cases may be due to deterioration caused by underlying diseases due to drug ineffectiveness. Therefore, the results of this study should be carefully interpreted.

## 4. Materials and Methods

### 4.1. Data Collection and Processing

This study is a case-non-case study using data from a national pharmacovigilance database. From January 2009 to December 2018, AE reports concerning antibacterial drugs for systemic use (anatomical therapeutic chemical (ATC) code J01) in the KIDS-KD were examined. The study protocol was exempted from review by the institutional review board of Ehwa Womans University (ewha-202004-0034-01).

The data consist of ‘patient information’, ‘drug information’, ‘AE information’, ‘seriousness of AE’, ‘reporter information’, ‘causality assessment’, and ‘past medical history’. All drugs were coded using the ATC code. AE information was coded using the PTs of the World Health Organization-Adverse Reactions Terminology (WHO-ART) version 092 [29]. Serious AEs were defined as a fatal or life-threatening AE; an AE resulting in persistent or significant disability, congenital abnormality or birth defect; an AE requiring hospitalization or prolongation of ongoing hospitalization; or other medically important conditions [30].

The data manager of KIDS extracted all reports, including ATC code requested by the researcher, and provided them in a form that can be downloaded by the researcher. First, the completeness of the data was reviewed through detection of input or logical errors. In order to eliminate duplicate cases, only the initial report was included in the study; follow-up reports were excluded from the analysis. Reports recorded as ’unlikely’ in the causal assessment item were excluded. Reports on post-operative pain, drug prescribing error, and medical device complications were also excluded. Some reports contained more than one AE or more than one drug. For analysis, one-to-one combinations of drug-AEs were made.

### 4.2. Statistical Analysis

Characteristics of AE reports about cephalosporins and all other systemic antibiotics (all other J01) were analyzed descriptively. These characteristics include patient demographics (sex and age), type of report, reporter, and seriousness of AE. The type of AE was analyzed at both PTs levels and SOC levels. 

Disproportionality analysis, which compares the observed count for a study drug–AE combination with an expected count, is a primary tool to detect signals [31]. There were three main indices, PRR, ROR, and IC, used in this study. A two-by-two contingency table was used to calculate these indices (Table 5) [32,33]. The subtle differences in chemical structure and pharmacokinetics between cephalosporin antibiotics can affect AEs [16]. Therefore, disproportionality analysis was conducted on individual cephalosporin antibiotics. Comparator was defined as all other J01. In this study, a drug–AE combination that met the criteria of all three indices was defined as a signal (Table 6). Among the signals, we focused on serious adverse events that were clinically meaningful.

In addition, ROR was calculated for major SOCs to evaluate differences in AEs by cephalosporin generations: body as a whole, general disorders (WHO-ART: 1810); cardiovascular disorders, general (WHO-ART: 1010); central and peripheral nervous system disorders (WHO-ART: 0410); and respiratory system disorders (WHO-ART: 1100).

The signals were compared with ADRs listed on the drug labels from the United States (USA), United Kingdom (U.K.), Japan, and South Korea. A signal that is not currently included on the drug labels was defined as a new signal. The label for each cephalosporin antibiotic was found through searching websites that are operated by drug regulatory authorities: ‘https://www.accessdata.fda.gov/scripts/cder/daf/’ (accessed on 10 May 2020) for the USA Food and Drug Administration (FDA), ‘https://products.mhra.gov.uk/’ (accessed on 10 May 2020) for U.K. Medicines and Healthcare Products Regulatory Agency (MHRA), ‘https://www.pmda.go.jp/index.html’ (accessed on 10 May 2020) for Japan Pharmaceuticals and Medical Devices Agency (PMDA), and ‘https://nedrug.mfds.go.kr/searchDrug’ (accessed on 10 May 2020) for Korea Ministry of Food and Drug Safety (MFDS). Drug databases such as DailyMed (https://dailymed.nlm.nih.gov/dailymed/; accessed on 10 May 2020) and Drugs.com (https://www.drugs.com/; accessed on 10 May 2020) were also used.

All statistical analyses were performed using SAS^®^ University Edition (SAS Institute, Cary, NC, USA) and Microsoft Excel 2019 (Microsoft, Redmond, WA, USA). A *p*-value less than 0.05 was considered statistically significant.

## 5. Conclusions

Detecting signals using a national pharmacovigilance database is useful for identifying unknown ADRs. Cephalosporins are relatively safe medications, but we identified some serious AEs that were not listed on drug labels. These AEs need to be monitored carefully in a clinical setting. This study identified signals of cephalosporins that warrant further investigation using other large databases, such as FDA Adverse Event Reporting System (FAERS). If causality with drugs is revealed, measures such as inclusion on drug labels or alerts will be needed.

## Figures and Tables

**Table 1:** Characteristics of AE reports.

Characteristics		Cephalosporins (*N* = 110,965)		All Other J01 (*N* = 45,780)	
		*N*	%	*N*	%
Sex	Male	48,035	43.29	22,973	50.18
	Female	61,851	55.74	22,299	48.71
	Unknown	1079	0.97	508	1.11
Age	≤10 years	6060	5.46	2190	4.78
	11–20 years	5544	5.00	1556	3.40
	21–30 years	9531	8.59	2418	5.28
	31–40 years	13,039	11.75	3499	7.64
	41–50 years	16,096	14.51	5239	11.44
	51–60 years	20,531	18.50	7356	16.07
	61–70 years	17,001	15.32	8209	17.93
	>70 years	16,790	15.13	10,625	23.21
	Unknown	6373	5.74	4688	10.24
Type of report	Spontaneous	83,781	75.50	24,413	53.33
	Post-marketing surveillance study	11,997	10.81	13,401	29.27
	Literature	193	0.17	372	0.81
	Others	14,994	13.51	7594	16.59
Reporter	Physicians	27,877	25.12	21,457	46.87
	Pharmacists	13,301	11.99	3272	7.15
	Nurses	53,814	48.50	12,847	28.06
	Consumers	771	0.69	125	0.27
	Public health centers	5176	4.66	2316	5.06
	Others	10,026	9.04	5763	12.59
Seriousness	Serious	7500	6.76	7004	15.30
	Non-serious	103,465	93.24	38,776	84.70
Type of seriousness ^1^	Death	509	6.34	2030	26.20
	Life-threatening	437	5.44	216	2.79
	Hospitalization/Prolonged	3911	48.71	3578	46.19
	Disabling	64	0.80	70	0.90
	Congenital anomaly	1	0.01	-	0
	Others	3107	38.70	1853	23.92

^1^ Duplicates allowed.

**Table 2:** Frequency of the 25 most commonly reported cephalosporin-induced AEs.

System Organ Classes	ADR (PT Code)	Cephalosporins (*N* = 163,800)		All Other J01 (*N* = 72,265)	
		*N*	%	*N*	%
Skin and appendage disorders	Rash	19,640	11.99	7152	9.90
	Pruritus	15,149	9.25	4050	5.61
	Urticaria	12,717	7.76	2857	3.95
	Skin reaction localized	1727	1.05	31	0.04
	Angioedema	1221	0.75	219	0.30
Gastrointestinal system disorders	Nausea	20,385	12.44	4988	6.90
	Vomiting	10,040	6.13	2270	3.14
	Diarrhea	8451	5.16	4202	5.81
	Diarrhea, *C. Difficile*	2902	1.77	3928	5.44
	Dyspepsia	2563	1.56	748	1.04
	Abdominal pain	2067	1.26	944	1.30
Body as a whole, general disorders	Fever	3170	1.94	2606	3.61
	Allergy	3087	1.88	421	0.58
	Chest pain	1584	0.97	483	0.67
	Anaphylactic reaction	1162	0.71	155	0.21
Central and peripheral nervous system disorders	Dizziness	5291	3.23	1231	1.70
	Headache	1834	1.12	746	1.03
Respiratory system disorders	Dyspnea	3138	1.92	1010	1.40
	Coughing	1001	0.61	563	0.78
White cell and reticuloendothelial system disorders	Granulocytopenia	1248	0.76	1251	1.73
	Leucopenia	1190	0.73	913	1.26
Liver and biliary system disorders	Hepatic enzymes increased	2286	1.40	791	1.09
Application site disorders	Application site reaction	1417	0.87	38	0.05
Psychiatric disorders	Somnolence	1159	0.71	143	0.20
Cardiovascular disorders, general	Hypotension	1087	0.66	538	0.74

ADR, adverse drug reaction; *C. Difficile, Clostridium difficile*; PT, preferred term.

**Table 3:** Serious AEs unlisted on the drug labels.

System Organ Classes	ADR (PT Code)	Drug (Number of Cases)
Central and peripheral nervous system disorders	Stupor	Cefaclor (10)
	Paralysis	Cefaclor (1)
Respiratory system disorders	Respiratory depression	Cefaclor (4)
Body as a whole, general disorders	Temperature changed sensation	Cefaclor (2)
	Chest pain	Cefoxitin (1)
Skin and appendage disorders	Skin discoloration	Cefotaxime (1)

ADR, adverse drug reaction; PT, preferred term.

**Table 4:** ROR (95% CI) of serious AEs by cephalosporin generation: disproportionality analysis compared with all other J01.

System Organ Classes	1st Generation	2nd Generation	3rd Generation	4th Generation
Body as a whole, general disorders	1.41 (1.06–1.88) ^†^	1.85 (1.52–2.26) ^†^	1.11 (0.88–1.4)	0.29 (0.15–0.56) ^†^
Cardiovascular disorders, general	2.03 (1.19–3.48) ^†^	1.61 (1.06–2.46) ^†^	1.06 (0.64–1.73)	0.17 (0.02–1.25)
Central and peripheral nervous system disorders	1.92 (1.03–3.56) ^†^	2.00 (1.25–3.19) ^†^	1.31 (0.76–2.24)	4.43 (2.34–8.40) ^†^
Respiratory system disorders	4.26 (2.64–6.90) ^†^	5.46 (3.66–8.13) ^†^	1.68 (1.05–2.69) ^†^	0.55 (0.17–1.84)

^†^*p* < 0.05.

**Table 5:** Two-by-two contingency table for disproportionality analysis.

Number of Cases	Each Cephalosporin	All Other J01
Specific adverse event	A	C
All other adverse events	B	D

**Table 6:** Disproportionality analysis and signal detection criteria.

Measures	Calculation	Criteria
PRR	(A / (A + B)) / (C / (C + D))	PRR ≥ 2, χ^2^ ≥ 4, A ≥ 3
ROR	(A / B) / (C / D)	ROR ≥ 2, χ^2^ ≥ 4, A ≥ 3
IC	Log_2_[A × (A + B + C + D) / ((A + B) × (A + C))]	Lower limit of 95% CI ≥ 0

CI, confidence interval; IC, information components; PRR, proportional reporting ratio; ROR, reporting odds ratio.

## Data Availability

The data presented in this study are available on request from the corresponding author. The data are not publicly available due to privacy.

## Supplementary Materials

The following are available online at https://www.mdpi.com/article/10.3390/ph14050425/s1, Table S1: 472 cephalosporin–AE combinations that satisfied all three criteria of the signal.
