# Supplementary material for: Signal Detection of Adverse Drug Reactions of Cephalosporins Using Data from a National Pharmacovigilance Database
Source: Pharmaceuticals (Basel). 2021 May 2;14(5):425. doi: 10.3390/ph14050425 (PMC8147424; doi:10.3390/ph14050425)
Supplement: Supplementary file 1 [file pharmaceuticals-14-00425-s001.zip › pharmaceuticals-1185662-supplementary.pdf]

Table S1. 472 cephalosporin–AE combinations that satisfied all three criteria of the signal.

Drug	PT	PRR	ROR	IC
cefatrizine	CORNEAL OEDEMA	440.64024	481.67333	3.8397972
cefatrizine	CORNEAL ULCERATION	346.21733	399.70171	4.4013435
cefbuperazone	SKIN REACTION LOCALISED	222.94905	246.42111	4.2500092
cefodizime	VEIN PAIN	105.50662	108.97476	4.3933428
cefotiam	APPLICATION SITE REACTION	103.03606	108.88114	3.6552805
cefoxitin	APPLICATION SITE REACTION	93.132743	97.87712	4.3789006
cefazedone	SKIN REACTION LOCALISED	89.550284	93.087837	2.4089007
cefroxadine	CRUSTING	82.777778	83.343714	3.5575346
cefmetazole	ERYTHEMA INDURATUM	80.503156	80.591822	2.4428785
cefradine	NEUROLOGIC DISORDER NOS	60.28782	60.386908	2.4937999
cefpiramide	DRUG REACTION PARADOXICAL	59.160868	59.496089	3.5429999
cefotiam	SKIN REACTION LOCALISED	57.688233	59.126688	3.5008826
cefpodoxime	INGROWING NAILS	48.44581	48.477639	2.2549677
cefaclor	ANAPHYLACTOID REACTION	43.186255	43.236737	2.2278987
cefmetazole	SKIN REACTION LOCALISED	42.415642	43.183178	3.8748356
cefcapene	PEPTIC ULCER	40.997542	41.088514	2.483683
cefetamet	PALMAR-PLANTAR ERYTHRODYSAESTHESIA	40.897001	41.609447	3.100091
cefdinir	FAECES DISCOLOURED	38.623731	38.684154	2.4256852
cefotaxime	APPLICATION SITE REACTION	37.247243	37.971371	2.767364
cefotetan	APPLICATION SITE REACTION	36.586014	37.284064	2.7604235
cefoxitin	INJECTION SITE REACTION	36.151527	37.521816	3.9324705
cefuroxime	CONCUSSION	34.765074	34.797592	2.3079594
cefradine	NAIL DISORDER	33.493233	33.674963	3.3439615
cefazolin	APPLICATION SITE REACTION	28.289909	28.702005	2.6059576
cefcapene	MOUTH DRY	28.159928	28.876882	3.9090795
cefuroxime	SOMNOLENCE	28.131738	29.754847	3.6793096
cefroxadine	APPLICATION SITE OEDEMA	26.607143	26.673311	2.4100547
cefroxadine	SKIN ULCERATION	26.607143	26.739822	2.9919594
cefcapene	SOMNOLENCE	26.389222	27.808747	4.0143953
cefuroxime	APPLICATION SITE REACTION	23.176716	23.450325	3.4014535
cefradine	ECZEMA	22.966788	23.015767	2.5525458
ceftriaxone	SKIN REACTION LOCALISED	21.618557	21.811561	1.5467862

cefpiramide	INJECTION SITE REACTION	21.534556	22.003986	3.6311834
cefuroxime	DRUG PRESCRIBING ERROR	21.393892	21.472683	3.0265081
cefaclor	ANAPHYLACTIC REACTION	20.556038	21.458039	2.2289806
cefaclor	SOMNOLENCE	19.988121	20.781482	2.220876
cefoperazone and beta-lactamase inhibitor	SKIN REACTION LOCALISED	19.507356	19.663536	3.2364885
flomoxef	SKIN REACTION LOCALISED	19.384831	19.538995	2.9473889
cefditoren	PULMONARY DISORDERS	19.270667	19.319519	2.8173578
cefetamet	MOUTH DRY	19.209197	19.534361	2.819901
cefminox	SKIN REACTION LOCALISED	18.559945	18.700875	3.1287713
cefoxitin	SKIN REACTION LOCALISED	17.492674	17.61737	3.1527719
cefadroxil	HYPERCHOLESTEROLAEMIA	17.384767	17.576178	3.264933
cefroxadine	EYE PAIN	17.24537	17.315454	2.6693317
cefuroxime	MOUTH DRY	17.206956	17.465718	3.2352149
cefcapene	DRUG PRESCRIBING ERROR	16.819504	16.867515	2.4821347
ceftriaxone	APPLICATION SITE REACTION	16.813169	16.954222	1.5086422
cefdinir	ECZEMA	16.553027	16.578006	2.217833
cefotiam	INJECTION SITE RASH	16.440262	16.759349	2.9499378
cefradine	OVARIAN HYPERSTIMULATION	15.458415	15.498734	2.5533425
cefaclor	TONGUE PARALYSIS	14.395418	14.400757	1.7899575
cefditoren	CRUSTING	14.274568	14.290319	2.2497194
cefotaxime	SKIN REACTION LOCALISED	13.814445	13.890837	2.3841158
cefazolin	SKIN REACTION LOCALISED	13.699932	13.77501	2.3173251
cefotiam	ALLERGY	13.676716	14.774211	2.8588984
cefradine	FLATULENCE	13.397293	13.438802	2.5858873
cefaclor	ANAPHYLACTIC SHOCK	13.329091	13.411505	2.0688298
cefazolin	ANAPHYLACTOID REACTION	13.271809	13.276318	1.8073019
cefuroxime	THIRST	13.243838	13.259565	2.2363777
cefaclor	APATHY	13.024426	13.039616	1.9681157
ceftezole	APPLICATION SITE REACTION	13.00969	13.092414	2.9099267
flomoxef	HEPATIC ENZYMES INCREASED	12.94738	14.920138	2.7888059
cefprozil	SOMNOLENCE	12.794579	13.105352	3.1887028
cefbuperazone	ALLERGY	12.650497	13.577442	3.0127377
cefixime	OESOPHAGITIS	12.473103	12.486982	2.0723825
cefixime	BURN	12.473103	12.486982	2.0723825
cefodizime	INJECTION SITE REACTION	12.379443	12.527549	2.6717469
cefetamet	SOMNOLENCE	12.240895	12.523939	2.7731884

ceftizoxime	INJECTION SITE URTICARIA	12.042661	12.055558	2.1723667
cefoperazone and beta-lactamase inhibitor	APPLICATION SITE REACTION	11.935422	12.004488	2.8102006
cefprozil	BRONCHITIS	11.87812	12.09514	3.0364655
cefroxadine	PERIODONTAL DESTRUCTION	11.640625	11.695758	2.559821
cefbuperazone	FLUSHING	11.615833	11.748871	2.8496749
cefozopran	GRANULOCYTOPENIA	11.553157	14.191447	2.2028319
cefaclor	LARYNX OEDEMA	11.036487	11.051839	1.9243806
cefotiam	INJECTION SITE REACTION	11.019035	11.134939	2.6426298
cefcapene	TASTE PERVERSION	10.932678	10.955269	2.087738
ceftezole	MALAISE	10.847482	11.037322	2.9257138
cefadroxil	FACE OEDEMA	10.830864	11.000229	2.9638607
cefditoren	FIXED ERUPTION	10.705926	10.720326	2.2338004
cefpodoxime	OTITIS MEDIA	10.62946	10.742671	2.6893752
cefodizime	INJECTION SITE RASH	10.432115	10.554876	2.5499072
cefuroxime	FLATULENCE	10.300763	10.324687	2.431731
cefroxadine	SKIN EXFOLIATION	9.897343	9.9822266	2.70131
flomoxef	VEIN PAIN	9.8798897	9.906679	2.3941897
cefroxadine	APPLICATION SITE REACTION	9.8026316	9.8482411	2.440426
cefmenoxime	HEPATIC ENZYMES INCREASED	9.7884685	10.843085	2.1157737
cefaclor	FLATULENCE	9.5969456	9.6175453	1.8951601
cefaclor	EYELID SKIN DISORDER	9.5969456	9.600372	1.6633156
cefpodoxime	CRYING ABNORMAL	9.2277733	9.2351344	1.9790552
cefotiam	INJECTION SITE PAIN	9.1678155	9.3079954	2.5153865
cefaclor	ANGIOEDEMA	9.136818	9.3685365	1.9218607
cefixime	TOOTH CARIES	8.9550482	8.9678841	2.0794634
cefradine	ANAPHYLACTIC REACTION	8.8162833	8.9669373	2.7613456
cefcapene	GASTRIC ULCER	8.7461422	8.769651	2.139958
cefprozil	FACE OEDEMA	8.5998118	8.7034027	2.6067785
cefixime	RASH PUSTULAR	8.5599725	8.5752267	2.1598327
cefalexin	LACERATION	8.5436453	8.5695982	2.2916899
cefalexin	GASTRIC ULCER	8.2588571	8.2796562	2.1852407
cefepime	APHASIA	8.1775489	8.183239	1.8886397
ceftizoxime	SKIN REACTION LOCALISED	8.1579319	8.1830695	2.3522909
cefdinir	URINARY INCONTINENCE	8.1313117	8.1465904	2.058279
cefpodoxime	OTITIS MEDIA CHRONIC	8.0743017	8.0822148	2.0003077
cefpodoxime	FLATULENCE	8.0743017	8.088558	2.1951832
cefminox	ALLERGY	8.0632158	8.4113609	2.7737848

cefradine	OEDEMA PERIORBITAL	8.038376	8.1015713	2.5433084
cefoperazone and beta-lactamase inhibitor	VEIN PAIN	8.0172277	8.0343967	2.2225234
cefamandole	SWEATING INCREASED	7.9804974	8.1632329	2.2724783
cefotetan	SKIN REACTION LOCALISED	7.9417222	7.9654522	2.0402992
cefaclor	FACE OEDEMA	7.9408355	8.0281034	1.8437256
cefepime	ENCEPHALOPATHY	7.8368177	7.8546583	2.0637125
cefradine	PALMAR-PLANTAR ERYTHRODYSAESTHESIA	7.7790735	7.8017713	2.2168326
cefradine	VAGINAL HAEMORRHAGE	7.7292077	7.7668431	2.3886065
cefprozil	GASTROENTERITIS	7.6820453	7.7148809	2.086354
cefoperazone	HYPOTENSION	7.6755178	8.0800946	2.1349993
cefcapene	OEDEMA PERIORBITAL	7.516216	7.5708905	2.4294429
cefazolin	HYPOTONIA	7.4653926	7.4707403	1.8009703
cefazedone	ALLERGY	7.4359792	7.7274133	1.7885774
cefuroxime	VEIN PAIN	7.3744096	7.3887525	2.1417792
cefaclor	OEDEMA GENERALISED	7.309823	7.3788638	1.7965952
cefaclor	FIXED ERUPTION	7.1977092	7.2038883	1.6877155
cefroxadine	FACE OEDEMA	7.1423304	7.2117051	2.4344849
cefradine	ANGIOEDEMA	6.9739335	7.1029161	2.5142319
cefmetazole	FLUSHING	6.9699702	7.0146389	2.3630091
cefadroxil	SOMNOLENCE	6.9059489	6.9889364	2.453169
cefadroxil	ANGIOEDEMA	6.8586479	6.9830059	2.5051933
cefaclor	DRUG REACTION PARADOXICAL	6.8549611	6.8588514	1.6133492
cefaclor	CRYING ABNORMAL	6.8549611	6.8588514	1.6133492
cefpodoxime	ASTHMA	6.8509226	6.8692846	2.1616542
cefbuperazone	VERTIGO	6.7759025	6.7824087	1.8477686
cefuroxime	LARYNGITIS	6.728724	6.7453076	2.1405848
cefprozil	OTITIS MEDIA	6.7096345	6.7518239	2.1630104
cefradine	ANAPHYLACTIC SHOCK	6.6986466	6.7177269	2.0967306
cefazedone	FLUSHING	6.6963577	6.737294	1.7166997
cefepime	APPLICATION SITE REACTION	6.6711583	6.6911227	1.973975
cefpodoxime	GASTROENTERITIS	6.6700753	6.694252	2.1842914
cefalexin	PALMAR-PLANTAR ERYTHRODYSAESTHESIA	6.6603687	6.6765875	2.025213
cefpodoxime	BRONCHITIS	6.6494249	6.7119694	2.2652442
cefazolin	TACHYCARDIA SUPRAVENTRICULAR	6.6359045	6.6395295	1.68443
cefodizime	TEMPERATURE CHANGED SENSATION	6.5848102	6.6574976	2.1705065

cefcapene	CATARACT	6.5075463	6.5284557	2.0325109
cefalexin	ARTHRITIS	6.352967	6.3652304	1.8957341
cefprozil	CONJUNCTIVITIS	6.3102515	6.356086	2.1625236
cefcapene	FACE OEDEMA	6.2887084	6.3412319	2.2883354
cefpiramide	SWEATING INCREASED	6.2746375	6.3825979	2.3902248
cefalexin	FACE OEDEMA	6.2124147	6.2635463	2.291864
cefbuperazone	ANAPHYLACTIC SHOCK	6.2112439	6.2274188	2.0886236
cefpodoxime	NERVOUSNESS	6.2110013	6.2285271	2.0867171
cefpodoxime	CREATINE PHOSPHOKINASE INCREASED	6.2110013	6.2168301	1.8274909
cefoperazone and beta-lactamase inhibitor	MALAISE	6.1992622	6.2560748	2.2783831
cefadroxil	ANAPHYLACTIC SHOCK	6.1812505	6.1972544	1.8993079
ceftezole	HEPATIC ENZYMES INCREASED	6.1763857	6.5517128	2.379523
cefditoren	SKIN ULCERATION	6.117672	6.1237445	1.7879257
cefuroxime	OEDEMA PERIORBITAL	6.0838879	6.1183605	2.2005045
cefminox	INJECTION SITE PAIN	6.056403	6.1133994	2.2676029
cefodizime	SWEATING INCREASED	6.0289496	6.1277718	2.2186514
cefditoren	SPUTUM DISORDER	6.001807	6.0572494	2.221506
flomoxef	INJECTION SITE REACTION	5.9666785	5.9976263	2.0658088
cefadroxil	DIABETES MELLITUS	5.9182186	5.9372225	1.9553494
cefaclor	DRUG PRESCRIBING ERROR	5.9058126	5.9110302	1.5846171
cefpirome	ALLERGY	5.8186722	5.9877485	2.3063826
cefozopran	NAUSEA	5.7939467	8.9899111	2.0807039
cefotiam	ALLERGIC REACTION	5.7804774	5.8093298	2.0487628
cefaclor	OEDEMA PHARYNX	5.7581673	5.7600638	1.439179
cefmetazole	DIZZINESS	5.7548966	6.2716907	2.2866223
cefadroxil	DYSPEPSIA	5.7267468	6.024586	2.3627153
cefroxadine	MOUTH DRY	5.6439394	5.6680013	1.9855591
cefditoren	GASTROESOPHAGEAL REFLUX	5.6346979	5.6484711	1.9908377
cefaclor	PARALYSIS	5.5982182	5.604639	1.5730857
cefadroxil	OEDEMA PERIORBITAL	5.5631255	5.591402	2.0490883
cefadroxil	SKIN DISORDER	5.5631255	5.5843001	1.9645414
cefaclor	OEDEMA PERIORBITAL	5.5182437	5.5460148	1.6166742
cefpodoxime	INJECTION SITE INFLAMMATION	5.5052057	5.5203577	1.9758127
flomoxef	MALAISE	5.4796027	5.5228143	2.0072214
cefprozil	SINUSITIS	5.4645477	5.4975369	1.9754808
cefpirome	TEMPERATURE CHANGED SENSATION	5.4292132	5.4766352	1.9259547

cefaclor	HYPOTONIA	5.3982819	5.4009119	1.4689593
cefadroxil	ANAPHYLACTIC REACTION	5.3836698	5.4348809	2.1460683
cefepime	STUPOR	5.3577045	5.3859948	1.8284176
cefditoren	INJECTION SITE INFLAMMATION	5.352963	5.3671968	1.9608635
ceftizoxime	INJECTION SITE PRURITUS	5.2686643	5.2773963	1.8464775
cefmetazole	NAUSEA	5.2281064	7.6160897	2.1796478
cefoperazone	HEPATIC ENZYMES INCREASED	5.2205165	5.4763054	1.8258497
cefepime	DYSKINESIA	5.2038948	5.2072274	1.6074654
flomoxef	INJECTION SITE PAIN	5.1912025	5.2316321	1.9541731
cefprozil	INSOMNIA	5.1249004	5.2335866	2.1671958
flomoxef	VASCULITIS	5.1142958	5.1186681	1.68372
cefroxadine	BULLOUS ERUPTION	5.1127451	5.1376277	1.9372237
cefpodoxime	GASTROESOPHAGEAL REFLUX	5.099559	5.1105817	1.8716794
ceftriaxone	VEIN PAIN	5.0770854	5.0833969	1.1495281
cefradine	FACE OEDEMA	4.9795249	5.0107544	2.0167598
cefaclor	SYNCOPE	4.9549447	4.9800513	1.5440115
cefotetan	VEIN PAIN	4.9370332	4.9429594	1.635227
cefditoren	MOUTH DRY	4.86633	4.8835904	1.9135987
ceftazidime	APPLICATION SITE REACTION	4.8411978	4.8510013	1.8166595
cefotetan	ANAPHYLACTIC SHOCK	4.8273213	4.8365475	1.6548125
cefalexin	DYSPEPSIA	4.802945	5.0018964	2.111887
cefaclor	TASTE PERVERSION	4.7984728	4.8022599	1.4498871
cefaclor	RESPIRATORY DEPRESSION	4.7984728	4.8009967	1.4107628
cefcapene	MEDICAL DEVICE COMPLICATION	4.7950341	4.809442	1.7689358
cefadroxil	DERMATITIS	4.7683933	4.78588	1.8191548
flomoxef	ALLERGY	4.7680786	4.875738	1.8922842
cefixime	CHRONIC OBSTRUCTIVE AIRWAYS DISEASE	4.6941785	4.7016324	1.6974162
cefixime	PALMAR-PLANTAR ERYTHRODYSAESTHESIA	4.6941785	4.7016324	1.6974162
ceftezole	SKIN REACTION LOCALISED	4.6904005	4.6978408	1.6966242
cefoperazone and beta-lactamase inhibitor	HEPATIC ENZYMES INCREASED	4.6826286	4.8815798	2.02245
cefaclor	DYSPEPSIA	4.6509262	4.8355736	1.5111263
cefpirome	COLITIS PSEUDOMEMBRANOUS	4.604626	4.6986597	1.9807242
cefazedone	APPLICATION SITE PAIN	4.6037459	4.606733	1.3931883
cefradine	DERMATITIS	4.5933577	4.6094174	1.8282946

cefminox	DIZZINESS	4.5336924	4.8294371	2.0720885
cefditoren	BRONCHITIS	4.4982882	4.5243948	1.8805755
ceftizoxime	COLITIS PSEUDOMEMBRANOUS	4.4895887	4.5782893	1.9307781
cefpiramide	VOMITING	4.4859099	5.0579805	2.0422715
cefazedone	INJECTION SITE REACTION	4.4809794	4.4972436	1.4495534
cefaclor	ALLERGIC REACTION	4.4785746	4.4948188	1.4667535
ceftriaxone	INJECTION SITE REACTION	4.4678352	4.4839902	1.1027906
cefroxadine	CONJUNCTIVITIS	4.4345238	4.4553031	1.7928801
cefditoren	ERYTHEMA MULTIFORME	4.4300383	4.436147	1.6566237
cefixime	CYSTITIS	4.4096828	4.4165627	1.6419765
cefpiramide	PARAESTHESIA	4.3701747	4.4112267	1.9185622
cefradine	ACNE	4.3581556	4.3750497	1.7972046
cefaclor	DYSPNOEA	4.3376294	4.5530288	1.4596174
cefotetan	ALLERGY	4.3342695	4.4206424	1.6196871
cefuroxime	FACE OEDEMA	4.3071773	4.3296024	1.8311137
cefotaxime	VEIN PAIN	4.2890765	4.2933768	1.5105819
ceftezole	POST-OPERATIVE PAIN	4.2765416	4.2831475	1.6142252
cefuroxime	ANGIOEDEMA	4.2331901	4.2752069	1.852777
cefpodoxime	SINUSITIS	4.1620112	4.1797753	1.7437173
cefadroxil	OEDEMA GENERALISED	4.1593462	4.1789238	1.7479373
cefotetan	INJECTION SITE REACTION	4.1514963	4.1651337	1.5535983
cefditoren	LARYNGITIS	4.1442294	4.1498291	1.5954911
cefaclor	DERMATITIS	4.1129767	4.1254286	1.4003472
cefepime	TENESMUS	4.0887745	4.093325	1.5071117
cefcapene	OEDEMA GENERALISED	4.0869823	4.1057768	1.7279473
cefradine	DYSPEPSIA	4.0836669	4.2197637	1.8995584
cefoxitin	ALLERGY	4.0675552	4.1420105	1.8499662
cefixime	EATING DISORDER	4.0422092	4.0483477	1.5626177
cefuroxime	ANAPHYLACTIC REACTION	4.0372344	4.0637647	1.7763363
cefmetazole	INJECTION SITE PAIN	4.0352459	4.0579563	1.7733596
cefprozil	UPPER RESPIRATORY TRACT INFECTION	4.0156146	4.0605121	1.8001939
cefroxadine	RASH ERYTHEMATOUS	3.9677318	4.0117174	1.8113627
cefradine	OEDEMA	3.9651182	4.0018483	1.8030519
cefepime	SKIN REACTION LOCALISED	3.9568785	3.9619061	1.4915598
cefalexin	MALAISE	3.9327891	3.9530385	1.7320296
cefazedone	INJECTION SITE RASH	3.9312887	3.9455501	1.3531644
cefmetazole	INJECTION SITE RASH	3.9196293	3.9337917	1.6967331
ceftizoxime	PARAESTHESIA	3.9184036	3.9502376	1.7445526
ceftriaxone	INJECTION SITE PAIN	3.9135549	3.9346926	1.0385021
cefuroxime	OEDEMA GENERALISED	3.8988868	3.915719	1.7060375

cefditoren	CYSTITIS	3.893064	3.8982164	1.5368439
cefodizime	PARAESTHESIA	3.8880161	3.9192717	1.6035616
cefminox	INJECTION SITE RASH	3.8788199	3.8926382	1.6143777
cefpirome	PARAESTHESIA	3.8468295	3.8773095	1.5924976
cefcapene	DYSPEPSIA	3.8001286	3.9147796	1.8197087
cefalexin	ANGIOEDEMA	3.7711676	3.8032043	1.7367588
cefpodoxime	ENTERITIS	3.7680074	3.7723441	1.4967611
cefaclor	OEDEMA MOUTH	3.7553265	3.7586237	1.2890024
cefalexin	FLUSHING	3.754026	3.7650863	1.5930156
cefamandole	CHEST PAIN	3.731771	3.8032834	1.5721038
cefoperazone and beta-lactamase inhibitor	GRANULOCYTOPENIA	3.7060338	3.8915443	1.7388628
ceftizoxime	MALAISE	3.6797021	3.6970057	1.6429506
cefminox	NAUSEA	3.6788794	4.5908991	1.8090344
cefazedone	DIZZINESS	3.676265	3.8550586	1.3139087
cefuroxime	DYSPEPSIA	3.6562199	3.7606979	1.7060673
cefazedone	NAUSEA	3.6394416	4.5251693	1.3066368
cefpodoxime	UPPER RESPIRATORY TRACT INFECTION	3.6089682	3.6438253	1.6216486
cefaclor	BRONCHOSPASM	3.5988546	3.600927	1.2300299
cefditoren	PHARYNGITIS	3.5910863	3.6539958	1.6668368
cefpodoxime	MUSCLE WEAKNESS	3.5885785	3.5920539	1.4228388
cefprozil	OEDEMA	3.5654336	3.5939736	1.6171871
cefdinir	PHARYNGITIS	3.562776	3.6244958	1.7050432
cefazedone	VOMITING	3.5394718	3.8572926	1.2837338
cefoxitin	INJECTION SITE RASH	3.5274997	3.538528	1.565587
cefditoren	OTITIS MEDIA	3.5234693	3.5332269	1.5516955
cefpiramide	NAUSEA	3.5195321	4.3282237	1.7281884
cefprozil	PHARYNGITIS	3.5189243	3.5788236	1.6880842
ceftizoxime	ANAPHYLACTIC SHOCK	3.5124429	3.5168468	1.4358226
cefprozil	RHINITIS	3.5121277	3.5526963	1.6486946
cefoxitin	SWEATING INCREASED	3.4594763	3.4869784	1.6206988
cefditoren	EPISTAXIS	3.4258963	3.4345525	1.512308
cefpodoxime	MOUTH DRY	3.4254613	3.4330732	1.4891839
cefditoren	UPPER RESPIRATORY TRACT INFECTION	3.406431	3.436755	1.586305
cefroxadine	DYSPEPSIA	3.4029635	3.4906941	1.6737936
cefotiam	SKIN DISCOLOURATION	3.3869985	3.3955315	1.471855
cefradine	OEDEMA GENERALISED	3.3806254	3.3926017	1.5184448
cefadroxil	ARTHRALGIA	3.3602771	3.3767442	1.5298492

cefixime	FACE OEDEMA	3.348237	3.3605962	1.5266835
cefotiam	FLUSHING	3.3430115	3.3513873	1.4578173
cefazolin	ALLERGY	3.3415956	3.3880854	1.3493809
cefepime	CONVULSIONS GRAND MAL	3.331594	3.3344999	1.3092695
cefradine	SOMNOLENCE	3.3262245	3.3418543	1.5356895
cefixime	OTITIS MEDIA	3.3156349	3.3240588	1.4725783
cefditoren	GASTROENTERITIS	3.2583253	3.263019	1.388446
cefmenoxime	RASH	3.2473138	4.3118309	1.5369023
cefpodoxime	EPISTAXIS	3.2297207	3.2372197	1.4310758
cefaclor	BRONCHITIS	3.1855408	3.1970659	1.1982277
cefadroxil	TEMPERATURE CHANGED SENSATION	3.1563833	3.1697458	1.4408136
cefpodoxime	PHARYNGITIS	3.1484698	3.1940672	1.4705552
cefalexin	OEDEMA	3.1479949	3.1690658	1.5029548
cefditoren	RHINITIS	3.1430241	3.1739424	1.4928763
cefpodoxime	EATING DISORDER	3.1400062	3.1433589	1.3183293
cefroxadine	PARAESTHESIA	3.1197655	3.1381343	1.4613614
cefditoren	COUGHING	3.1186001	3.171356	1.4945074
ceftezole	INJECTION SITE REACTION	3.1019182	3.1087068	1.3829165
cefixime	MOUTH DRY	3.086778	3.0926776	1.3584863
cefroxadine	OEDEMA	3.0624066	3.0820826	1.4499545
flomoxef	PARAESTHESIA	3.0197475	3.0366839	1.3725778
cefmetazole	OEDEMA PERIORBITAL	3.0188684	3.025638	1.3613836
cefaclor	OEDEMA	3.0124941	3.0313781	1.1547967
cefoxitin	SKIN DISCOLOURATION	3.004282	3.0106347	1.346088
cefpodoxime	DERMATITIS	2.9990263	3.0048505	1.3380358
cefbuperazone	RASH ERYTHEMATOUS	2.9803782	3.0023448	1.4161925
cefamandole	URTICARIA	2.9692145	3.2310176	1.5036664
cefuroxime	PALPITATION	2.9479156	2.9662029	1.4062533
cefoperazone and beta-lactamase inhibitor	FLUSHING	2.9451041	2.9512272	1.328466
cefroxadine	CONSTIPATION	2.9439795	3.002471	1.4756877
cefodizime	ALLERGY	2.9404853	2.9743064	1.3850104
cefodizime	VOMITING	2.9299651	3.1256927	1.5020432
ceftezole	ANGIOEDEMA	2.921327	2.9384887	1.4023822
cefdinir	BRONCHITIS	2.9211225	2.9304083	1.3486021
cefamandole	DYSPNOEA	2.920388	3.0021066	1.3854297
cefpodoxime	RHINITIS	2.9136624	2.9392299	1.3706838
cefradine	OEDEMA PERIPHERAL	2.8896123	2.9012439	1.359623
cefditoren	HAEMOPTYSIS	2.8723216	2.8784439	1.3064584

cefixime	UPPER RESPIRATORY TRACT INFECTION	2.8662938	2.886043	1.3868881
cefdinir	DYSPEPSIA	2.8399802	2.8957065	1.4182899
cefditoren	GASTRITIS	2.8339216	2.8388251	1.2713138
cefprozil	ANGIOEDEMA	2.8237655	2.839507	1.3077812
cefdinir	ANGIOEDEMA	2.8218251	2.8375389	1.3593983
cefodizime	CHEST PAIN	2.8192052	2.8549538	1.3488761
cefaclor	STUPOR	2.8128978	2.819058	1.0836535
cefazolin	HYPERLIPAEMIA	2.8074981	2.8093257	1.1052291
cefazolin	INJECTION SITE PAIN	2.7940651	2.8033384	1.1618825
cefazolin	NAUSEA	2.793225	3.2216405	1.1771729
cefpirome	AGRANULOCYTOSIS	2.7837057	2.8183782	1.333163
cefmetazole	SOMNOLENCE	2.7759709	2.7859185	1.3107359
cefotetan	VOMITING	2.7707676	2.9396621	1.1884177
ceftezole	SWEATING INCREASED	2.7695698	2.7853759	1.3363031
ceftezole	VOMITING	2.7659112	2.9340191	1.3800898
cefamandole	VOMITING	2.7599659	2.9271135	1.3875316
cefotetan	URTICARIA	2.7483279	2.961366	1.1801427
cefminox	SWEATING INCREASED	2.7398013	2.7551731	1.3112354
ceftizoxime	SWEATING INCREASED	2.7369685	2.7522992	1.3073957
cefotetan	NAUSEA	2.7315149	3.1339243	1.1740811
flomoxef	INJECTION SITE RASH	2.7295399	2.7353736	1.2263615
cefotiam	SWEATING INCREASED	2.6978689	2.7126386	1.2507688
cefpodoxime	SKIN DISORDER	2.6914339	2.6952221	1.196351
cefpodoxime	PARONYCHIA	2.6914339	2.6956019	1.2044754
cefaclor	INSOMNIA	2.6908563	2.7139595	1.0550668
cefotaxime	ALLERGY	2.6895872	2.7164826	1.1531724
ceftizoxime	NAUSEA	2.66971	3.0469917	1.3097996
ceftizoxime	VOMITING	2.6633319	2.8152657	1.3047251
cefbuperazone	OEDEMA GENERALISED	2.6597	2.666262	1.2319225
cefaclor	TEMPERATURE CHANGED SENSATION	2.6544743	2.6630879	1.0365328
cefazolin	INJECTION SITE REACTION	2.6543618	2.6589319	1.0977636
cefditoren	SINUSITIS	2.6488889	2.6547725	1.2206819
ceftizoxime	PRURITUS	2.6479883	2.9352575	1.2989516
ceftezole	DIZZINESS	2.6458279	2.723507	1.3172265
ceftriaxone	FLUSHING	2.6400844	2.6447111	0.8028461
cefepime	AGRANULOCYTOSIS	2.6267278	2.6565331	1.1522874
cefuroxime	CONJUNCTIVITIS	2.6014681	2.6071379	1.199192
cefmetazole	PALPITATION	2.5892828	2.6023731	1.2467079
cefpodoxime	FACE OEDEMA	2.5723439	2.578694	1.184583
cefotetan	ANAPHYLACTIC REACTION	2.5693807	2.5780775	1.0949531

cefalexin	SOMNOLENCE	2.5630936	2.5711739	1.1978947
cefbuperazone	URTICARIA	2.5623161	2.7383517	1.2526849
cefazedone	ANAPHYLACTIC SHOCK	2.5576366	2.5596238	0.9661533
ceftezole	ALLERGY	2.5557669	2.5792814	1.2537554
cefoperazone and beta-lactamase inhibitor	INJECTION SITE RASH	2.5480114	2.5528844	1.1635618
cefaclor	URTICARIA	2.5437282	2.7162666	1.0059244
cefotiam	DIZZINESS	2.5423124	2.6121291	1.196729
cefotaxime	DIARRHOEA	2.5409712	2.8081213	1.0979947
cefotetan	OEDEMA PERIORBITAL	2.5343437	2.5386606	1.0668001
cefazedone	SWEATING INCREASED	2.5310638	2.5435522	0.985682
cefixime	DYSPEPSIA	2.5290828	2.5701872	1.2517243
cefotetan	TEMPERATURE CHANGED SENSATION	2.5163696	2.5238515	1.0713643
cefoperazone and beta-lactamase inhibitor	SWEATING INCREASED	2.5087923	2.52099	1.2046906
cefoxitin	URTICARIA	2.508302	2.6742747	1.250897
cefadroxil	OEDEMA	2.4946751	2.5062707	1.1861944
cefmetazole	VOMITING	2.4931992	2.6201392	1.2379558
cefazolin	VOMITING	2.4866379	2.6126593	1.0566437
cefcapene	UPPER RESPIRATORY TRACT INFECTION	2.4846995	2.4982998	1.1953845
cefprozil	DYSPEPSIA	2.4802325	2.5192349	1.2392528
cefradine	ABDOMINAL PAIN	2.472035	2.5211029	1.2380438
cefditoren	FACE OEDEMA	2.4633104	2.4689686	1.1408785
cefditoren	CELLULITIS	2.4534414	2.458194	1.1245934
cefotetan	INJECTION SITE PAIN	2.4499563	2.4565238	1.0418615
cefazolin	SKIN DISCOLOURATION	2.4448069	2.4485313	1.0132355
cefditoren	FATIGUE	2.4417024	2.4472771	1.1304018
ceftriaxone	OEDEMA MOUTH	2.4281713	2.4292759	0.7329469
cefradine	DYSPNOEA	2.4274304	2.4775589	1.2145793
cefazolin	DIZZINESS	2.4204071	2.4814896	1.0274559
cefetamet	ABDOMINAL PAIN	2.4199922	2.4662963	1.163943
cefradine	RASH ERYTHEMATOUS	2.4175567	2.4302845	1.1674334
cefminox	PRURITUS	2.414488	2.6359087	1.2315379
cefotiam	NAUSEA	2.4141445	2.6969733	1.1367053
cefotetan	MALAISE	2.4136607	2.4196351	1.0249321
cefazolin	SWEATING INCREASED	2.4130562	2.4240405	1.0170894
ceftriaxone	URTICARIA	2.4036515	2.5509879	0.7449832

cefixime	ANGIOEDEMA	2.3921019	2.4022674	1.1437549
cefazedone	INJECTION SITE PAIN	2.3884095	2.3945396	0.9272475
ceftriaxone	ANAPHYLACTIC SHOCK	2.3580241	2.3596213	0.7194315
cefaclor	CONJUNCTIVITIS	2.3502724	2.3545898	0.9173933
cefbuperazone	PRURITUS	2.3483997	2.5528218	1.141374
cefpiramide	CHEST PAIN	2.3472733	2.369244	1.1539549
cefpodoxime	SOMNOLENCE	2.3387632	2.3450753	1.0785865
cefadroxil	PHARYNGITIS	2.3325474	2.3533847	1.1440379
cefpodoxime	DYSPEPSIA	2.3316165	2.3645487	1.1057243
flomoxef	FLUSHING	2.3246799	2.3279693	1.0299539
cefalexin	ABDOMINAL PAIN	2.3208877	2.3621412	1.1535452
cefbuperazone	ANGIOEDEMA	2.3205145	2.3298666	1.0946319
cefixime	URINARY TRACT INFECTION	2.2976768	2.3039788	1.0715006
cefpiramide	DIZZINESS	2.2876176	2.3398287	1.1379057
cefoperazone and beta-lactamase inhibitor	PAIN	2.2718608	2.2767577	1.0440942
cefroxadine	DIZZINESS	2.2694963	2.3205485	1.1343404
cefbuperazone	HYPOTENSION	2.2670306	2.2887822	1.0840183
cefetamet	DIZZINESS	2.2657802	2.3165962	1.0962293
cefazolin	MALAISE	2.2646341	2.2696474	0.9426333
ceftazidime	RASH ERYTHEMATOUS	2.2634172	2.2740316	1.0573373
cefotaxime	ANGIOEDEMA	2.2537613	2.2623834	0.958385
cefoxitin	CHEST PAIN	2.2532115	2.2728165	1.0909845
ceftriaxone	MALAISE	2.2339176	2.2387425	0.6897691
cefbuperazone	ANAPHYLACTIC REACTION	2.2294905	2.2353982	1.0308926
ceftizoxime	DIZZINESS	2.2255934	2.273889	1.0701211
cefaclor	NERVOUSNESS	2.2146797	2.2161333	0.8406606
cefadroxil	RHINITIS	2.2116401	2.2238884	1.0536153
cefazedone	URTICARIA	2.2051556	2.3202127	0.8594205
cefotetan	DIZZINESS	2.1999409	2.2466594	0.9395815
cefaclor	PARAESTHESIA	2.1942765	2.2015368	0.8571122
cefaclor	GASTROENTERITIS	2.1906071	2.1922697	0.8347329
cefamandole	PRURITUS	2.1843435	2.3496007	1.0883304
flomoxef	VOMITING	2.1788116	2.2654593	1.0032954
cefotiam	PARAESTHESIA	2.1785719	2.1856851	0.9886384
ceftriaxone	ALLERGY	2.1755492	2.1906397	0.6730122
cefodizime	NAUSEA	2.1711792	2.3776843	1.095901
cefmetazole	ALLERGY	2.1671475	2.1820716	1.0341314
cefotetan	FLUSHING	2.1628907	2.1655769	0.8952949

cefoxitin	RASH	2.1625959	2.4792426	1.0556383
ceftriaxone	VOMITING	2.1562076	2.240248	0.6673234
cefodizime	DIZZINESS	2.1369876	2.1799404	1.0395816
cefotetan	PRURITUS	2.127072	2.2796562	0.903408
cefdinir	RHINITIS	2.1260769	2.1370156	1.0067373
ceftizoxime	URTICARIA	2.1104174	2.2114608	1.0041839
ceftezole	NAUSEA	2.1042402	2.2919194	1.0192344
ceftazidime	PARAESTHESIA	2.1010224	2.1074296	0.956737
ceftriaxone	ANGIOEDEMA	2.0809095	2.0877692	0.6392218
cefpodoxime	SPUTUM DISORDER	2.0797444	2.0838618	0.9294137
cefazolin	OEDEMA PERIORBITAL	2.0737202	2.0761908	0.8394516
cefaclor	INJECTION SITE INFLAMMATION	2.0720678	2.073422	0.7788853
cefixime	COUGHING	2.0677731	2.0852561	0.9801445
cefaclor	DIZZINESS	2.0542609	2.0924908	0.7966036
cefmetazole	HEADACHE	2.0503485	2.0730609	0.9702403
cefotetan	ANGIOEDEMA	2.0499584	2.0565219	0.8531726
cefbuperazone	CIRCULATORY FAILURE	2.0464135	2.0508475	0.920416
flomoxef	DIZZINESS	2.0461338	2.0839135	0.9240863
ceftezole	PARAESTHESIA	2.0458631	2.0517887	0.9355922
ceftriaxone	PARAESTHESIA	2.0430804	2.0489821	0.6252786
cefbuperazone	PARAESTHESIA	2.0429857	2.0488866	0.928829
ceftazidime	RIGORS	2.0426428	2.0504219	0.9274943
cefradine	URTICARIA	2.0405541	2.1318308	0.9880684
cefditoren	MYALGIA	2.039224	2.0491717	0.9417201
ceftriaxone	OEDEMA PERIORBITAL	2.0384498	2.0407985	0.6204005
cefditoren	DYSPEPSIA	2.0324084	2.0545939	0.9487359
cefazolin	RASH ERYTHEMATOUS	2.0207078	2.0283567	0.8269484
cefdinir	URTICARIA	2.0150336	2.1028617	0.9696648
cefuroxime	DIZZINESS	2.014548	2.0506014	0.9449011
cefadroxil	ABDOMINAL PAIN	2.0057929	2.032826	0.9586182
cefotaxime	SKIN DISCOLOURATION	2.0056208	2.0077464	0.8120775
ceftizoxime	ALLERGY	2.0023427	2.0141733	0.921412
cefminox	VOMITING	2.0014665	2.0686847	0.967829
cefditoren	CONSTIPATION	2.0008286	2.0210993	0.9278209
